# Smoking shapes the relationship between metabolic biomarkers and femoral-neck bone mineral density: A stratified analysis by smoking status

**DOI:** 10.18332/tid/221015

**Published:** 2026-08-01

**Authors:** Amal M. Hassan, Saja A. Abdallah, Amal Al-Haidose, Mohammed Al-Hamdani, Atiyeh M. Abdallah

**Affiliations:** 1 Department of Biomedical Sciences, College of Health Sciences, QU Health, Qatar University, Doha, Qatar; 2 University of Birmingham Medical School, Birmingham, United Kingdom; 3 Department of Public Health, College of Health Sciences, QU Health, Qatar University, Doha, Qatar

**Keywords:** biomarkers, low bone density, femoral neck, smoking, Qatar biobank

## Abstract

**INTRODUCTION:**

Bone mineral density (BMD) is a crucial indicator of bone strength and osteoporosis risk. Smoking is known to negatively impact bone health and related bone metabolism biomarkers. However, if and how smoking status influences the relationship between bone metabolism biomarkers and BMD, particularly measured at the femoral neck, remains poorly understood. The objective of this study was to examine the relationship between bone metabolism biomarkers and BMD stratified by smoking status.

**METHODS:**

Data from 4464 healthy individuals were collected from the Qatar Biobank. The sample was divided into smokers, non-smokers, and ex-smokers, and logistic regression was used to examine relationships between biochemical markers and femoral-neck BMD in the different subgroups.

**RESULTS:**

Sex, age, and body mass index consistently influenced BMD across all smoking strata. Males and older individuals were at higher risk of low BMD, while higher BMI was protective. Creatinine levels were positively associated with BMD in all groups. Alkaline phosphate (ALP) was significantly associated with BMD in non-smokers and ex-smokers but not in smokers, while cholesterol levels were negatively associated with BMD in non-smokers and smokers but not in ex-smokers.

**CONCLUSIONS:**

Smoking status impacts the relationship between metabolism-related biomarkers and BMD, highlighting the need for smoking status-specific bone health guidelines. These findings emphasize the importance of including smoking history in clinical bone health assessments and suggest that personalized approaches may be necessary to mitigate the risk of bone deterioration.

## INTRODUCTION

Bone mineral density (BMD) is the most important diagnostic measure of bone strength and osteoporosis, the latter characterized by reduced bone mass and an increased risk of fracture^1^. The increasing prevalence of osteoporosis in the aging population mandates the study of intrinsic and environmental BMD-related factors, together with knowledge of risk factors, to guide prevention and treatment^1^. BMD is usually measured at the femoral neck, as this site is prone to osteoporotic fractures^2^ and femoral-neck fractures are associated with loss of quality of life, significant morbidity, and a high mortality rate within the first year after the injury^3,4^. Furthermore, the management of hip fractures, particularly subcapital fractures, is expensive and incurs a significant burden on healthcare systems. Several biomarkers, such as calcium, phosphorus, creatinine, alkaline phosphatase (ALP), parathyroid hormone (PTH), and vitamin D, are known to be associated with BMD and fracture risk through their participation in bone metabolism^4,5^ and, as the femoral neck contains a high proportion of trabecular bone, it is particularly responsive and sensitive to changes in overall bone metabolism, making it an excellent candidate for studies seeking to establish relationships between active bone metabolism and bone health.

Smoking tobacco has an overall negative effect on bone health. Smokers typically have a lower body mass index (BMI) and are less physically active, contributing to bone loss with age^6^. Nicotine has been shown to decrease osteoblast number, increase healing times, increase oxidative stress, and reduce calcium absorption, impairing osteoblast function. Furthermore, smoking increases bone resorption^7^. Smoking is associated with a higher risk of fracture^8^, and after fracture, smoking is a significant risk factor in femoral-neck fixation failure^7^. Therefore, smoking not only accelerates bone loss and risk of injury but also increases the recovery period after the injury has been sustained^8^.

Several studies have examined the relationship between smoking, BMD, and bone metabolism biomarkers. Meravi et al.^9^ reported that, in addition to postmenopausal women having a lower BMD than premenopausal women, smoking and other lifestyle choices aggravate bone loss. Ucar et al.^5^ showed that the BMD of smokers was lower than that of non-smokers, especially in males and in the lumbar spine bodies and femoral neck. Ghadimi et al.^10^ found that elderly male smokers had low BMDs. These findings corroborate the hypothesis that smoking has a negative influence on bone health.

While densitometry is still the ‘gold standard’ diagnostic for osteoporosis, other laboratory-based markers also act as surrogates of bone metabolism prior to measurable changes in BMD^5^. For example, serum calcium, phosphorus, and vitamin D, together with enzymes like alkaline phosphatase and hormones like parathyroid hormone, reflect ongoing bone remodeling processes^5,11^. These biomarkers have complex relationships with BMD and can be modified through lifestyle exposures and comorbidities, and understanding these relationships is important for tailoring bone health advice.

Despite the evidence that tobacco smoking has a negative impact on bone health, the relationships between smoking, bone metabolism factors, and BMD remain incompletely understood. Previous studies have not explored the interaction between smoking, bone metabolism biomarkers, and outcomes^2,5^, so they have failed to incorporate the modifying effects of smoking on bone health and its surrogates^12^. To address this gap, here we explored the effect of smoking on the relationship between bone metabolism biomarkers and femoral-neck BMD to enhance understanding of how clinical biomarkers and non-clinical extrinsic factors influence bone health.

## METHODS

### Study participants

This was a study of data from 4464 healthy individuals enrolled in the Qatar Biobank (QBB)^13^. Ethical approval was obtained from QBB (E-2021-QF-QBB-RES-ACC-00050-0172) and Qatar University (QU-IRB 1648-E/22). This study formed part of a larger analysis to identify clinical and non-clinical factors related to femoral neck BMD, extending the analysis by examining these relationships for different smoking strata. The QBB cohort provides a comprehensive dataset representing the Qatari population. Key exclusion criteria were diagnoses of colon or rectal polyps, Crohn’s disease, thyroid disease, and cancers (mouth, bladder, thyroid, Hodgkin lymphoma, pharyngeal, esophagus, stomach bowel cancer, gallbladder, larynx, breast, and prostate/cervical cancer) or a history of bowel surgery. Secondary data were requested from the QBB according to the above exclusion criteria and specifying variables of interest.

To detect an odds ratio of 1.3 with 80% power at a 5% significance level (two-tailed), assuming the baseline probability of the outcome is 0.2, a total sample size of 721 participants was needed for the smallest stratum, in this case, ex-smokers.

### Variables

Study variables included demographic variables (age and sex), anthropometric measurements (BMI), and biochemical markers relevant to bone metabolism (calcium, creatinine, total cholesterol, ALP, magnesium, uric acid, fibrinogen, testosterone, and estradiol), chosen because of their known or hypothesized contribution to bone health. Calcium and ALP, for instance, directly affect bone formation and turnover^14^, while creatinine is a proxy for muscle mass, which can influence mechanical loading on bones^15^. Cholesterol, testosterone, and estrogen have been shown to affect bone physiology via lipid metabolism and hormonal regulation^16,17^.

Data were collected according to standardized QBB protocols. Demographic information was self-reported and cross-checked against participant records. BMI was calculated from height and weight measurements recorded during clinical screening. Fasting venous blood samples were collected, and assays were performed in approved facilities. Calcium, creatinine, ALP, cholesterol, uric acid, and magnesium levels were determined using photometric and enzymatic colorimetric assays, while fibrinogen was measured using coagulation-based techniques. Testosterone and estradiol levels were measured using immunoassays. Participants were categorized into non-smokers, current smokers, and past smokers.

### BMD measurements

BMD measurements were recorded at the femoral neck, a clinically relevant region prone to osteoporotic fractures, using the Lunar Prodigy dual-energy X-ray absorptiometry (DXA) device (GE Healthcare, Madison, WI, USA). This device is widely used in clinical and research settings for its high-resolution imaging and precise quantification of bone mineral content and density, with reliable reproducibility across repeated scans^18^. BMD values were dichotomized and classified as normal or low according to conventional diagnostic standards. The femoral neck was chosen for BMD measurements, as it is the usual site for fractures in osteoporotic individuals, and femoral-neck BMD is frequently used as a baseline for determining fracture risk and commencing treatment.

### Statistical analysis

Continuous variables are presented as means with standard deviations (SD), and categorical variables are summarized as frequencies and percentages. Following stratification by smoking status, two-tailed multivariate logistic regression analysis was used to compute adjusted odds ratio (AOR). All variables were selected based on biological relevance, as noted above, and also informed by a prior study. Through this analysis, a follow-up to a previous study reporting the relationship between demographic and biomarker variables and femoral-neck BMD in a non-stratified analysis^19,20^, we examined the relationship between biochemical markers and femoral-neck BMD. All analyses were performed using SPPS v.29 (IBM Statistics, Armonk, NY). A p<0.05 was considered significant.

## RESULTS

### Sample characteristics

Data for 4464 QBB participants were collected for the period 2017–2022. Of these 4464 participants, 2414 were male (54.1%), and 2050 were female (45.9%); 61.2% were non-smokers, 21.7% were smokers, and 17.1% were ex-smokers (Table 1).

**Table 1:** Demographic and clinical characteristics of healthy participants, by smoking status, a cross-sectional study of data from the Qatar Biobank, 2017–2022 (N=4464)

*Characteristics*	*Non-smokers* *(N=2732)* *Mean (SD)*	*Smokers* *(N=970)* *Mean (SD)*	*Ex-smokers* *(N=762)* *Mean (SD)*
**Age** (years)	34.64 (10.68)	33.67 (8.89)	33.43 (10.16)
**Measurements**			
BMI (kg/m^2^)	28.39 (6.13)	27.80 (5.46)	27.63 (5.31)
Creatinine (µmol/L)	63.43 (13.84)	74.86 (12.36)	73.59 (12.73)
Alkaline phosphatase (U/L)	69.14 (19.25)	71.34 (18.35)	70.11 (18.92)
Total cholesterol (mmol/L)	4.81 (0.85)	4.88 (0.96)	4.84 (0.92)
Calcium (mmol/L)	2.36 (0.09)	2.39 (0.09)	2.38 (0.09)
Uric acid (µmol/L)	279.75 (78.57)	327.67 (70.10)	324.25 (80.75)
Magnesium (mmol/L)	0.83 (0.06)	0.84 (0.07)	0.83 (0.06)
Fibrinogen (g/L)	3.27 (0.65)	3.13 (0.61)	3.05 (0.60)
Testosterone (nmol/L)	6.79 (8.99)	18.08 (9.43)	15.01 (9.72)
Estradiol (pmol/L)	289.44 (327.12)	122.48 (118.03)	158.29 (186.41)
***Sex***	***n (%)***	***n (%)***	***n (%)***
Male	912 (33.4)	890 (91.8)	612 (80.3)
Female	1820 (66.6)	80 (8.2)	150 (19.7)

### Logistic regression analysis for femoral-neck BMD in non-smokers

The logistic regression model for non-smokers demonstrated a statistically significant effect on femoral-neck BMD (χ^2^=776.36, n=2732, p<0.001), with a Nagelkerke R^2^ of 0.436 and a correct classification of 85.2% of cases. The model showed that several factors had a significant impact on femoral-neck BMD in non-smokers, including sex (reduced likelihood of a normal femoral-neck BMD in males; AOR=0.023, 95% CI: 0.012–0.044), age (increasing age associated with a decreased likelihood of a normal BMD; AOR =0.953; 95% CI: 0.939–0.966), and BMI (likelihood of having a normal BMD increasing with BMI; AOR =1.180; 95% CI: 1.140–1.221). For biochemical parameters, creatinine had a significant positive effect on femoral-neck BMD (AOR=1.029; 95% CI: 1.015–1.043), while ALP and total cholesterol levels demonstrated a significant negative effect on BMD (ALP, AOR=0.992; 95% CI: 0.985– 0.999); total cholesterol, AOR=0.856; 95% CI: 0.733–0.999) (Table 2).

**Table 2:** Multivariate logistic regression analysis for predictors of femoral-neck BMD in non-smokers, a cross-sectional study of data from the Qatar Biobank, 2017–2022 (2017–2022) (N=2732)

*Predictors*	*Wald χ^2^*	*df*	*p*	*AOR*	*95% CI*	
					*Lower*	*Upper*
Sex	130.404	1	**<0.001**	0.023	0.012	0.044
Age	46.730	1	**<0.001**	0.953	0.939	0.966
BMI	88.901	1	**<0.001**	1.180	1.140	1.221
Creatinine	17.933	1	**<0.001**	1.029	1.015	1.043
ALP	5.434	1	**0.020**	0.992	0.985	0.999
Total cholesterol	3.881	1	**0.049**	0.856	0.733	0.999
Calcium	0.014	1	0.904	0.914	0.210	3.971
Uric acid	1.548	1	0.213	0.999	0.996	1.001
Magnesium	2.804	1	0.094	0.143	0.015	1.392
Fibrinogen	1.447	1	0.229	0.857	0.667	1.102
Total testosterone	0.385	1	0.535	1.007	0.985	1.029
Estradiol	0.258	1	0.612	1.000	0.999	1.001

Sex reference group: female. Smoking status reference group: non-smoker. Significant p-values (p<0.05) are bolded.

### Logistic regression analysis for femoral-neck BMD in smokers

For smokers, the regression model yielded a statistically significant effect on femoral-neck BMD (χ^2^=205.72, n=970, p<0.001), with a Nagelkerke R^2^ of 0.263 and a correct classification of 64.4%. Again, several demographic variables were associated with femoral-neck BMD: sex (reduced odds of a normal femoral-neck BMD in males; AOR=0.032; 95% CI: 0.008–0.134); age (AOR=0.954; 95% CI: 0.936–0.973); and BMI (AOR=1.171; 95% CI: 1.126– 1.218). Of the biomarkers, creatinine was significantly positively associated with femoral-neck BMD (AOR=1.034; 95% CI: 1.019–1.050), while cholesterol was negatively associated (AOR=0.793; 95% CI: 0.671–0.938) (Table 3).

**Table 3:** Multivariate logistic regression analysis for predictors of femoral-neck BMD in smokers, a cross-sectional study of data from the Qatar Biobank, 2017–2022 (N=970)

*Predictors*	*Wald χ^2^*	*df*	*p*	*AOR*	*95% CI*	
					*Lower*	*Upper*
Sex	22.259	1	**<0.001**	0.032	0.008	0.134
Age (years)	23.164	1	**<0.001**	0.954	0.936	0.973
BMI	61.520	1	**<0.001**	1.171	1.126	1.218
Creatinine	20.733	1	**<0.001**	1.034	1.019	1.050
ALP	3.145	1	0.076	0.992	0.984	1.001
Total cholesterol	7.342	1	**0.007**	0.793	0.671	0.938
Calcium	3.041	1	0.081	0.249	0.052	1.188
Uric acid	0.334	1	0.563	1.001	0.998	1.003
Magnesium	2.775	1	0.096	0.139	0.014	1.416
Fibrinogen	0.011	1	0.917	0.985	0.744	1.305
Total testosterone	0.461	1	0.497	1.007	0.986	1.029
Estradiol	1.772	1	0.183	1.002	0.999	1.006

Sex reference group: female. Smoking status reference group: non-smoker. Significant p-values (p<0.05) are bolded.

### Logistic regression analysis for femoral-neck BMD in ex-smokers

The logistic regression model for ex-smokers demonstrated a statistically significant effect on femoral-neck BMD (χ^2^=163.95, n=762, p<0.001), with Nagelkerke R^2^ of 0.275 and correct classification of 70.1%. As with non-smokers and smokers, male sex (AOR=0.048; 95% CI: 0.013–0.182), age (AOR=0.959; 95% CI: 0.941–0.978), and BMI (AOR=1.125; 95% CI: 1.074–1.179) were associated with femoral-neck BMD. For the biochemical parameters, there was a positive association between creatinine levels (AOR=1.030; 95% CI: 1.012–1.048) and ALP (AOR=0.982; 95% CI: 0.973–0.992) and femoral-neck BMD (Table 4).

**Table 4:** Multivariate logistic regression analysis for predictors of femoral-neck BMD in ex-smokers, a cross-sectional study of data from the Qatar Biobank, 2017–2022 (N=762)

*Predictors*	*Wald χ^2^*	*df*	*p*	*AOR*	*95% CI*	
					*Lower*	*Upper*
Sex	20.004	1	**<0.001**	0.048	0.013	0.182
Age	18.009	1	**<0.001**	0.959	0.941	0.978
BMI	24.425	1	**<0.001**	1.125	1.074	1.179
Creatinine	10.585	1	**<0.001**	1.030	1.012	1.048
ALP	12.537	1	**<0.001**	0.982	0.973	0.992
Total cholesterol	1.041	1	0.308	0.902	0.740	1.100
Calcium	2.553	1	0.110	4.959	0.696	35.363
Uric acid	0.463	1	0.496	0.999	0.996	1.002
Magnesium	1.511	1	0.219	0.152	0.008	3.065
Fibrinogen	1.159	1	0.282	0.840	0.612	1.154
Total testosterone	0.005	1	0.945	0.999	0.973	1.026
Estradiol	1.892	1	0.169	1.002	0.999	1.006

Sex reference group: female. Smoking status reference group: non-smoker. Significant p-values (p<0.05) are bolded.

**Figure 1:**
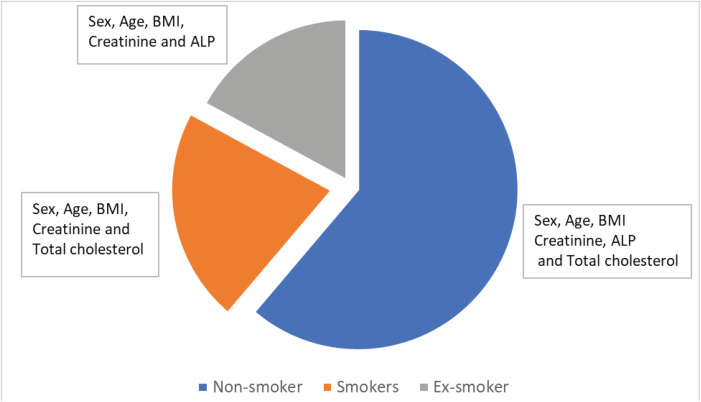
Significant correlates across smoking status strata, with text in each rectangle indicating the variables showing significant associations

Figure 1 summarizes all significant associations for each smoking status stratum.

## DISCUSSION

This study highlights key relationships between biochemical laboratory parameters and femoral-neck BMD according to past and current smoking status. In examining non-smokers, smokers, and ex-smokers separately, we discovered consistent influences of sex, age, BMI, and specific bone metabolism-related markers on femoral-neck BMD (creatinine, ALP, and cholesterol).

Sex, age, and BMI had a similar quantitative relationship with femoral-neck BMD regardless of smoking status. Several studies have reported comparable results on the influence of sex, age, and BMI on BMD in diverse populations. In the cross-sectional Korean National Health and Nutrition Examination Survey, age and BMI were significantly associated with lower BMD in both males and females^20^. Our data confirm that age-related bone loss and BMI-related mechanical stress are universal drivers of BMD. Sng et al.^21^ reported considerable variability in BMD in different ethnic and geographical groups, with Asian males having a lower peak BMD than their White counterparts. Population-specific differences in BMD must be considered to avoid underdiagnosis and misclassification, especially when using Western-based BMD reference values, and local, stratified bone health examinations are a requirement for diagnostic accuracy.

We detected sex-related differences in BMD, with males less likely to maintain normal femoral-neck BMD than females. The differences were most evident in non-smokers, underscoring the protective role of estrogen in non-smokers, which helps to maintain BMD and reduce fracture risk^22^. However, in our analysis, the protective effect of estrogen in females was less pronounced in smokers and ex-smokers, perhaps because smoking leads to an imbalance in bone turnover, increasing the risk of osteoporosis and fragility fractures^12^. Thus, sex differences in BMD are most pronounced when no additional factors, like smoking, compromise bone health^10^.

Sex-based differences in BMD are well-reported. Chen et al.^23^ recently evaluated over 15000 postmenopausal women and discovered that a menopause at a later age was strongly related to improved bone health, as determined by the quantitative ultrasound index (QUI)^23^. Their findings highlighted the protective impact of estrogen in preserving bone mass during and after menopause. Importantly, the study found that BMI partially mediated this link, supporting the hypothesis that both hormonal and mechanical variables play a role in maintaining bone density^24^. Although the study did not explicitly examine the effect of smoking, the findings support the concept that hormonal protection (particularly estrogen) is a crucial determinant of BMD.

Age was also a consistent predictor of BMD across all smoking status groups, with increasing age related to a slight decrease in the likelihood of maintaining normal BMD. This natural decrease in BMD is due to decreased bone formation and increased bone resorption, particularly after the age of 50years. Older adults, especially postmenopausal women, are at a higher risk of accelerated bone loss^25^. The universal decrease in BMD due to aging was present regardless of smoking status. Previous studies have suggested that smoking promotes bone resorption and delays fracture healing by altering osteoblast-osteoclast homeostasis^12^, especially in the bones of the lower body^26^.

Analysis of longitudinal data showed that bone loss during a seven-year period in the non-weight-bearing distal forearm was predictive of higher long-term mortality, particularly in males with normal baseline BMD^27^. While the study did not reveal a significant association between BMD loss and mortality in women, the findings highlight the clinical importance of age-related bone loss in predicting health outcomes other than fractures. Men with a >4% reduction in distal forearm BMD were 50% more likely to die than those with steady or increasing BMD^27^. These findings indicate that bone loss in elderly populations, particularly in less often examined skeletal areas, may be a helpful predictor of systemic health decline and should not be neglected in preventative care programs.

The positive association between BMI and BMD was consistent across all smoking groups, reinforcing the idea that a higher body weight is protective in terms of bone density. Rinonapoli et al.^28^ reported that a higher body weight protected against fractures and a lower BMI increased the risk of osteoporosis, with the strongest association between higher BMI and BMD occurring in non-smokers^28,29^. It is thought that the additional mechanical load occurring at higher weights more effectively promotes bone formation^30^. While smokers also benefited from a higher BMI, the effect was diminished, perhaps due to the oxidative stress and inflammation caused by smoking^6^. Ex-smokers also showed a positive BMI–BMD relationship, although again slightly attenuated, possibly due to residual metabolic changes from past smoking affecting bone metabolism even after cessation^31^. This highlights how the impact of BMI on BMD varies depending on an individual’s smoking history.

Jiao et al.^32^ reported that in fully adjusted models, total percent fat (TPF), android percent fat (APF), gynoid percent fat (GPF), and visceral adipose tissue percent (VAT%) were all negatively associated with BMD, whereas total lean mass percent (TLM%) was positively associated^32^. Therefore, not all increases in body mass increase BMD; rather, lean muscle mass may be the primary protective factor^33^. Furthermore, the study detected age- and sex-specific differences, including an inverted U-shaped association between BMD and APF in males and middle-aged adults, with peak bone density occurring at certain fat distribution thresholds^32^.

Creatinine levels were positively associated with BMD in all groups, indicating that greater muscle mass, as reflected by creatinine levels, supports bone density. Previous studies have demonstrated that muscle mass mechanically stresses bones to preserve or increase BMD^15^. This association was stronger in smokers, where muscle mass may counteract the detrimental effects of smoking on bone health. Conversely, non-smokers, who typically maintain better overall muscle and bone health, exhibited a less significant association between creatinine and BMD. Previous studies have shown that while muscle mass might increase BMD, smoking-related toxins compromise bone integrity, underscoring the beneficial role of muscle mass in smokers^30^.

ALP was significantly associated with BMD in non-smokers and ex-smokers but not in smokers. Increased circulating ALP levels may be a biomarker for osteoporosis treatment and detection and might also signal an increased risk of osteoporosis or a reduction in bone density^27^. The absence of the association in smokers may be due to the dominant effect of smoking on bone metabolism, disrupting or masking normal relationships between bone turnover markers and BMD^31^. Most current studies either evaluate ALP in general osteoporosis populations or ignore smoking as a stratifying variable, limiting the ability to make clear conclusions on the biomarker’s reliability in active smokers. Furthermore, current research frequently lacks longitudinal data and focuses on postmenopausal women, creating a gap in understanding of ALP’s biomarker potential in different populations of smokers, such as males and young individuals. Further research is now required to determine if smoking affects ALP’s utility as a diagnostic or prognostic marker of bone health.

Cholesterol was negatively associated with BMD in both non-smokers and smokers but not ex-smokers. Higher cholesterol levels are reported to be associated with lower BMD^16^. The negative influence of cholesterol in non-smokers suggests a lack of metabolic adaptation to mitigate its adverse effects on bone health, unlike in ex-smokers. Thorin et al.^25^ reported that quitting smoking may improve cholesterol metabolism and enhance bone quality, thus reducing the negative influence of cholesterol on BMD. Furthermore, Nakamura et al.^34^ revealed that current cigarette smoking is related to elevated triglyceride and sdLDL-C levels and lower HDL-C levels. The influence of smoking on lipid profiles appears to persist after quitting, further supporting the need for smoking cessation. Despite these associations, existing evidence on the interaction between cholesterol and BMD, particularly in the setting of smoking status, is limited and fragmented^34^. Most studies have focused on the influence of cholesterol on cardiovascular outcomes, with only a few expanding their study to include skeletal characteristics such as bone density. Even fewer studies stratified their findings by smoking, which is a significant confounding factor considering the independent effects of tobacco use on lipid metabolism and bone health. In addition, different studies have evaluated different lipid characteristics, with some concentrating on total cholesterol and others on LDL-C, HDL-C, or triglycerides, making comparisons difficult^34^. Longitudinal and mechanistic studies to assess lipid profiles, bone metabolism, and smoking status are urgently needed to better understand these interactions over time to better inform comprehensive, lifestyle-specific guidelines for osteoporosis prevention and treatment.

### Implications for clinical practice

The findings from this study significantly inform the clinical management of bone health, highlighting the subtle interacting effects of smoking on bone metabolism biomarkers and BMD. Healthcare providers are advised to incorporate a patient’s smoking history into the evaluation of BMD and related biochemical markers. This approach will ensure a more accurate assessment and enable personalized management strategies. Additionally, understanding the specific presence or alteration of bone health biomarkers across different smoking statuses can refine diagnostic processes and therapeutic interventions. These findings will allow clinicians to better address specific bone health challenges faced by patients based on their smoking history, enhancing the effectiveness of treatments and preventive measures in clinical practice.

### Strengths and limitations

A main strength of this investigation is the large sample size of 4464 participants from the Qatar Biobank, and using standardized data collection protocols ensured high quality of data collection. There are, however, some study limitations. First, the cross-sectional design does not allow for causal inference between biomarkers and bone density. Second, dependence on single-point measurements of biomarkers may not adequately measure long-term exposure. Since the cohort was obtained from only one biobank center, generalizability to other ethnic and geographical groups is potentially limited.

Since this study concentrated on cross-sectional data, longitudinal studies would be useful to provide further insights into the long-term consequences of smoking and the possible reversibility of its negative effects on bone health. Examining bone metabolism biomarkers over time in both smokers and ex-smokers may help to understand how quitting influences bone metabolism. Including other lifestyle elements like food and physical activity would help to provide a more comprehensive overview of BMD determinants in different populations. Research that compares the effects of smoking at other bone locations, such as the lumbar spine, would be beneficial for evaluating the site-specific effects of smoking. Clinically, healthcare professionals should consider smoking history in BMD assessments, specifically for individuals with or at risk of osteoporosis. Including smoking-specific changes in BMD diagnostic instruments might result in more individualized interventions. Targeted counseling and cessation programs can also minimize smoking-related bone loss, thereby promoting long-term bone health and reducing fracture risk in this population.

## CONCLUSIONS

This study shows that smoking significantly influences the relationship between bone metabolism biomarkers and femoral-neck BMD. Creatinine and BMI showed different associations with BMD depending on smoking status, highlighting the complexity of smoking’s effect on bone health. Smoking not only directly influences bone density but also alters how biochemical markers interact with bone health measurements. Generally, smokers had a lower BMD and displayed altered biochemical profiles.

These findings highlight the importance of clinical guidelines tailored to the specific needs of smokers when evaluating bone health. Integrating smoking history into diagnostic pathways could help to more accurately estimate osteoporosis risk and optimize treatments, considering the consistent, negative association between smoking and BMD. By targeting the distinct metabolic changes induced by smoking, personalized approaches, particularly for smokers and ex-smokers, may enhance clinical outcomes. Ultimately, BMD assessments and related healthcare decisions should take smoking histories into account. By improving bone health management and optimizing preventative measures, this customized strategy could possibly reduce osteoporosis risk in groups impacted by smoking.

## Data Availability

The data supporting this research are available from the following source: Qatar Biobank https://www.qphi.org.qa/.
